# Psychotic-Like Experiences at the Healthy End of the Psychosis Continuum

**DOI:** 10.3389/fpsyg.2017.00775

**Published:** 2017-05-15

**Authors:** Lui Unterrassner, Thomas A. Wyss, Diana Wotruba, Vladeta Ajdacic-Gross, Helene Haker, Wulf Rössler

**Affiliations:** ^1^Collegium Helveticum, ETH Zurich and University of ZurichZurich, Switzerland; ^2^Department of Psychiatry, Psychotherapy and Psychosomatics, Psychiatric Hospital, University of ZurichZurich, Switzerland; ^3^Translational Neuromodeling Unit, Institute for Biomedical Engineering, University of Zurich and ETH ZurichZurich, Switzerland; ^4^Department of Psychiatry and Psychotherapy, Charité Universitätsmedizin BerlinBerlin, Germany

**Keywords:** subclinical psychosis, psychosis continuum, healthy individuals, psychotic-like experiences, exceptional experiences, sex differences, exploratory factor analysis

## Abstract

There is increasing evidence pointing toward a continuous distribution of psychotic symptoms and accompanying factors between subclinical and clinical populations. However, for the construction of continuum models, a more detailed knowledge of different types of psychotic-like experiences (PLE) and their associations with distress, functional impairment, and demographic variables is needed. We investigated PLE in a sample of healthy adults (*N* = 206) incorporating the recently developed revised Exceptional Experiences Questionnaire (PAGE-R). For the first time, the PAGE-R was cross validated with PLE, disorganized-, and negative-like symptoms [Schizotypal Personality Questionnaire (SPQ), Physical Anhedonia Scale (PAS)]. We subjected the PAGE-R to exploratory factor analyses and examined the resulting subtypes of EE for specific associations with contextual factors, valence ratings, socio-demographic variables, and general psychological burden (Revised Symptom-Checklist-90). Correlational cross-validation suggested that the PAGE-R measures facets of PLE. Importantly, we (1) identified three types of exceptional experiences (EE): Odd beliefs, dissociative anomalous perceptions, and hallucinatory anomalous perceptions. Further, the results suggested that even in healthy individuals (2) PLE and EE are indicative of reduced functioning, as reflected by increased psychological burden and lower educational achievement. Moreover, (3) similar sex-differences might exist as in psychotic patients with women reporting more positive-like symptoms and EE but less disorganized-like symptoms than men. Importantly, (4) EE might be differentially implicated in psychological functioning. We suggest that the PAGE-R holds the potential to complement the current assessment of sub-clinical psychosis. However, whereas our results might point toward a continuity of psychotic symptoms with EE and normal experiences, they require replication in larger samples as well as equivalence testing across the psychosis continuum. Future analyses incorporating the PAGE-R might shed more light onto mechanisms that are implicated in the progress or resilience toward clinical illness.

## Introduction

Psychosis refers to delusions and hallucinations or “positive symptoms,” which have long been seen as key characteristics of psychotic disorders such as schizophrenia. However, it has become increasingly evident that psychotic symptoms are not restricted to individuals with a psychotic disorder. Accordingly, psychosis research has shifted from a categorical to a rather dimensional approach toward psychosis. The dimensional approach to psychosis includes concepts such as psychotic-like experiences (PLE), schizotypal symptoms, psychosis proneness, or at-risk mental states (Meehl, 1962; Claridge, 1997; Van Os et al., 2000; Yung et al., 2003). A recent meta analysis suggests that the prevalence of PLE or “positive-like symptoms” in the general population lies at ~7% (Linscott and van Os, 2013). In about 80% of cases these experiences are transitory, in about 20% they become persistent and in ~7% they predate the onset of a psychotic disorder (Kaymaz et al., 2012; Linscott and van Os, 2013; Zammit et al., 2013). Moreover, PLE and psychotic disorders have been shown to share etiological risk factors, cognitive correlates and demographic characteristics (van Os et al., 2009; Linscott and van Os, 2013). These findings have led to the suggestion that psychosis constitutes a phenotype that can be examined across a temporal and phenomenological continuum of increasing severity and persistence of symptoms between healthy and clinical populations (van Os and Linscott, 2012). However, constructs have not clearly been defined and there is no consensus of what exactly constitutes the subclinical psychosis phenotype (Kwapil and Barrantes-Vidal, 2015; Rössler et al., 2015).

The research on sub-clinical psychosis has mainly focused on PLE since they appear to be the best early indicators of psychosis onset (Tandon et al., 2012). However, in the literature on PLE there has been a tendency not to differentiate between different types of experiences (Yung et al., 2009). Further, little attention has been given to investigating whether the psychotic symptoms of healthy individuals are phenotypically similar to those of clinical populations (DeRosse and Karlsgodt, 2015). Moreover, whereas sex differences have been found regarding almost all aspects of schizophrenia, the results concerning PLE and associated disorganized- and negative-like symptoms appear to be more ambiguous with some studies finding differences (Bora and Baysan Arabaci, 2009) and others not (Rössler et al., 2012). A central question is also whether all types of PLE equally affect functioning and well-being, and are similarly implicated in the development of psychosis spectrum disorders (Brett et al., 2014). For example, Yung and Lin (2016) have suggested that paranormal and magical thinking are not necessarily maladaptive and might account for the observation that PLE in the general population do mostly not coincide with mental disorder. In contrast, those PLE that are associated with distress and poor functioning might be more indicative of vulnerability toward psychosis (Yung et al., 2009). Hence, before continuum models can be constructed, a more detailed knowledge of types of PLE, their prevalence and associations with demographic variables from healthy to clinical populations is needed (Scott et al., 2008). In this line of thought, new psychometric instruments have been demanded that have a high phenomenological resolution and capture already subtle signs of reality distortion (Heckers, 2008).

Regarding a comprehensive description of PLE, the investigation of exceptional experiences (EE) in healthy individuals is of interest. EE are defined as deviations from ordinary experiences, or experiences that are consistent with typical “reality models” (Metzinger, 2003). For example, dreaming an event prior to its occurrence might conflict with existing subjective concepts of cause and effect. EE include for example hearing voices of beloved dead ones, déjà-vus, or out-of-body experiences. We assessed EE in a sample of healthy adult subjects using the recently developed PAGE-R questionnaire (Fach et al., 2013; Landolt et al., 2014). The PAGE-R is a condensed version of the earlier PAGE questionnaire. The latter is based on a collection of EE that have been reported by individuals from the general population at the Institute for Institute for Frontier Areas of Psychology and Mental Health in Germany between 1996 and 2006 (IGPP; Fach et al., 2013). Although it has been suggested that some EE are conceptually close to PLE (Brugger and Graves, 1997) it is still unknown how they are exactly related. It has been speculated that EE might even belong to another category of phenomena than PLE (Landolt et al., 2014). We sought to close this knowledge gap by cross-validating EE with PLE, disorganized- and negative-like symptoms. We operationalized PLE by the Schizotypal Personality Questionnaire (SPQ) subscales paranormal beliefs, unusual perceptual experiences, ideas of reference, and suspiciousness (Raine, 1991; Raine et al., 1994) as well as the SCL-90-R subscales schizotypal signs and schizophrenia nuclear symptoms (SNS; Rössler et al., 2007). We measured disorganized-like symptoms using the SPQ subscales odd speech and odd behavior. Negative-like symptoms were captured using the SPQ subscales excessive social anxiety, constricted affect, no close friends, and the Physical Anhedonia Scale (PAS; Chapman et al., 1976). If EE represent PLE, we expected them to correlate more strongly with positive-like than with disorganized- or negative-like symptoms.

More recently, it has been proposed that the psychosis continuum needs to be accurately mapped out and new instruments are needed that capture already subtle signs of reality distortion (Heckers, 2008). We chose to implement the PAGE-R, as it differs in several aspects from many self-report instruments dealing with PLE and might therefore extend their current assessment. First, it includes a rich variety of 32 items rated on five-point Likert-scales that promise a fine-grained phenomenological description of EE. Second, its items have neither been created on the basis of clinical symptoms nor attenuated versions of them but represent exceptional experiences that have been reported by individuals from the general population (Fach et al., 2013). Therefore, the PAGE-R might tap experiences that are part of psychotic symptomatology but have mostly not been considered in research. For example, “psychotic-like” sleep-related experiences are also included that are often not assessed in research on (subclinical) psychosis (e.g., van Os et al., 2009). Third, in contrast to most surveys preoccupied with subclinical psychosis it not only assesses associated distress but also comfort that the experiences may confer. Fourth, many of the included EE have a rather enriching or neutral than distressing connotation (e.g., seeing meaning in coincidences). Research has often focused on negatively valenced PLE, which may have biased findings regarding their significance for psychological functioning (Wiseman and Watt, 2004; Perdue, 2013). Hence, assessing positive PLE might especially be important, as they might also be implicated in the maintenance of mental health (*cf*. McCreery and Claridge, 2002). Fifth, the PAGE-R also inquires into the context in which EE occurred. Assessing the context of occurrence is important to characterize subtypes of EE and to evaluate their significance for mental health. For example, a dissociative experience during meditation might have a different subjective meaning and appraisal than independent of that context. Lastly, the PAGE-R does not include beliefs in the paranormal, such as witchcraft or poltergeists, which might be less relevant for subclinical psychosis as they might be substantially influenced by cultural differences (Schulter and Papousek, 2008). Not discerning between beliefs and experiences might obscure specific associations between subtypes of PLE and other factors. Hence, if EE as measured by the PAGE-R can be reconciled with the concept of subclinical psychosis, this questionnaire might contribute to a more comprehensive and fine-grained description of PLE.

In order to advance our understanding of the complex architecture of psychotic disorder, the continuum from health to disease needs to be accurately mapped out. We aimed at exploring PLE at the healthy end of the psychosis continuum and incorporated a questionnaire assessing EE. We sought to (1) identify latent factors underlying EE, (2) cross-validate EE with measures for PLE, disorganized- and negative-like symptoms, (3) investigate distress and comfort associated with EE, (4) explore the associations of EE and PLE with general psychological burden, age, and educational achievement, and (5) test for specific sex differences in PLE, EE, as well as disorganized-, and negative-like symptoms. We hypothesized that EE reflect facets of PLE and complement the description of subclinical psychosis. Moreover, against the background of the continuum model of psychosis, we expected that EE in healthy individuals are also associated with distress. Further, we expected that PLE and EE in healthy people are indicative of reduced psychological functioning. The latter would for example be reflected in higher psychological burden and lower educational achievement. However, similar to Yung et al. (2006), we hypothesized that some experiences might be neutral or even beneficial to some degree for the maintenance of psychological functioning. If sex differences exist across the whole psychosis continuum, we expected more PLE and EE in women and more disorganized- and negative-like symptoms in men (see Bora and Baysan Arabaci, 2009; Ochoa et al., 2012). This research serves to refine dimensional models of psychosis and might help to uncover mechanisms that are implicated in the maintenance of mental health and the development of mental illness, respectively.

## Materials and methods

### Study design and sampling

The “Exceptional Experiences” project started with a large online survey (Fach et al., 2013; Landolt et al., 2014; *N* = 1580). Ninety-one subjects from the online survey agreed to take part in a three-level follow-up study at the Collegium Helveticum of the Federal Institute of Technology Zurich (ETHZ) and the University of Zurich (UZH). One hundred and forty-six additional participants were acquired by online ads, pamphlets and word-of-mouth. After a telephone screening eligible subjects underwent examinations in the first study level that included 14 self-report questionnaires and three visual perception tasks in randomized order and was concluded by a blood sampling. The following study levels encompassed cognitive, perceptual, behavioral, and neurobiological measurements (see Supplementary Figure 1, for the study overview). The present study is based on level 1 data.

### Inclusion/exclusion criteria

Inclusion criteria were age between 20 and 60 years and good command of the German language. Eighteen (7.6%) of the 237 acquired subjects were excluded from the analysis in order to meet the requirements of the multilevel study: A parent with history of psychosis (1), multiple sclerosis (1), Guillain-Barré-Syndrome (1), attention deficit hyperactivity disorder (3), craniocerebral trauma (1), current use of antidepressants (1), consumption of drugs other than alcohol, nicotine or cannabis <10 days before the examination or on a regular basis (3), insufficient German skills (1), epilepsy (2), panic attacks (1), and episodes of bodily numbing (1), and for practical reasons nicotine craving within 4 h of deprivation (2). The study sample was subclinical, as none of the participants reported a psychiatric diagnosis, however no formal diagnostic interviews were attained. In order to control for potential psychopathology, 13 other individuals were excluded on the basis of “caseness” criteria for psychoticism and paranoid ideation as operationalized by the SCL-90-R definition (Derogatis, 1983). The final sample (*M*_SPQ_ = 18.86, *SD* = 10.56, Range = 0–66) consisted of 73 women and 133 men (*M*_age_ = 33.11 years, *SD* = 11.23, Range = 20–60).

The study was conducted in accordance with principles enunciated in the “Declaration of Helsinki,” the guidelines of Good Clinical Practice (GCP) and Swiss regulatory authority's requirements. Volunteers gave fully written consent for the study, which was approved by the sub-commission of the ethics committee of the Canton of Zurich, specialized on psychiatry, neurology, neurosurgery and health care science (Department D; Prof. Dr. med. Dr. phil. Paul Hoff; KEK-ZH, Nr. 2011-0423).

### Measures

#### The SCL-90-R

The Symptom Checklist-90-Revised (SCL-90-R; Derogatis, 1977) assesses general and particular psychopathology in the last few weeks on five-point Likert-scales, ranging from 0 (*not at all*) to 4 (*extremely*). The global severity index (GSI) reflects the mean psychological distress across all 90 symptoms. The caseness criterion was used to control the population for the presence of psychotic symptoms that might be of clinical relevance. Accordingly, subjects that were above a cut-off of 63 in both, the t-transformed paranoid ideation subscale and the psychoticism subscale were removed from the analysis (Derogatis, 1977). In response to contradictory factor analytic results, (Rössler et al., 2007) modified the psychosis subscales on the basis of principal component analyses. The first scale “schizotypal signs” (STS) contains items that are reminiscent of criteria required for diagnosing schizotypal personality disorder, e.g., reduced capacity for close relationships, as well as ideas of reference, odd beliefs and suspicious-paranoid ideas. The second scale, “schizophrenia nuclear symptoms” (SNS) represents a collection of nuclear symptoms of schizophrenia such as thought insertion, thought broadcasting, thought control, and hearing voices. We implemented the STS and SNS as measures for PLE.

#### The PAGE-R

The PAGE-R assesses the frequency of 32 EE on five-point Likert-scales ranging from 0 (*never*) to 4 (*very often*). Nine items specify the circumstances under which the experiences occurred, two items assess their enriching and burdensome valence on five-point Likert-scales ranging from 0 (*not at all*) to 4 (*very much*). The latter valence items operationalized distress and comfort associated with EE. The contextual variables comprise: Wakefulness, occult practices (during automatic writing, commuting, séances), surprisingly, mental techniques (during meditation, yoga, autogenous training, etc.), contact with healers (therapists, healers, psychics, spiritual or religious groups), against own volition, drug-induced (under the influence of alcohol, drugs, psychoactive substances, medication), on own volition, and extreme (or life-threatening) situations. The questionnaire concludes with information on socio-economic status and psychosocial factors. Educational achievement was indicated on a five-point Likert-scale (0 = *no education*, 1 = *compulsory school*, 2 = *vocational training*, 3 = *high-school diploma*, 4 = *university or another type of higher education*).

#### The SPQ

PLE, disorganized-, and negative-like symptoms were detected using the SPQ (Raine, 1991). The SPQ comprises 74 forced-choice items (*yes* or *no*) distributed over nine subscales. These nine subscales depict four subtypes of positive-like symptoms (ideas of reference, paranormal beliefs, perceptual experiences, paranoid ideation), two subtypes of disorganized-like symptoms (odd behavior and odd speech), and three subtypes of negative-like symptoms (social anxiety, no close friends, constricted affect; Raine et al., 1994).

#### The PAS

The PAS (Chapman et al., 1976) was included in addition to the SPQ in order to account for non-social aspects of negative-like symptoms. Thirty forced-choice items (*yes* or *no*) assess deficits in the ability to experience pleasure in non-social contexts and from typically pleasurable physical stimuli such as food, sex, and settings.

### Statistical analysis

#### Missing data replacement

Missing data replacement for the ordinal PAGE-R and SCL-90-R items was carried out using the expectation maximization algorithm in the SPSS 21 missing value analysis procedure (IBM Corporation, 2012). Three cases were missing in the PAS data.

#### Factor analyses

In order to identify latent factors underlying EE, the 32 PAGE-R items were subjected to exploratory factor analyses for ordinal Likert-type data (Costello and Osborne, 2005; Baglin, 2014) using the stand-alone software FACTOR (Lorenzo-Seva and Ferrando, 2006; see Supplementary Data 1, for more statistical information). The eigenvalue criterion suggested the retention of 5 factors (Guttman, 1954; Kaiser, 1960). The eigenvalues of the first 5 factors were 15.29, 2.01, 1.47, 1.25, and 1.17 (see Supplementary Figure 2, for the scree plot). Both, the minimum average partial test (Velicer, 1976) and parallel analysis (Timmerman and Lorenzo-Seva, 2011) suggested the retention of two factors. After performing Promin rotation there was evidence for two factors of EE explaining 55.32% of common variance (see Supplementary Table 1, for the 2-factor solution of the PAGE-R items). Subsequently, a rotation on three factors was explored, which explained 60.02% of common variance and lead to a partitioning of the second factor into two subordinate factors displaying more homogeneous item content than the superordinate factor. In both analyses the communalities were above the minimum of 0.4 as recommended by Costello and Osborne (2005). As their specific associations with other variables would be more easily interpretable and the eigenvalue of the third factor was well above 1 (Guttman, 1954; Kaiser, 1960), subscales were created on the basis of the three factor solution. Items were included in a scale if they loaded over 0.32 on the respective factor. Four cross-loading items with very small loading differences (≤ 0.1) were not included in the subscales.

#### Regression analyses

As the context of occurrence of exceptional experiences was indicated with regard to all experiences in the PAGE-R questionnaire, we applied regression analyses in order to untangle specific associations between the identified subtypes of exceptional experiences with different contextual variables. In order to characterize the novel subtypes of exceptional experiences, the nine context variables of the PAGE-R were entered in hierarchical multiple linear regression models predicting the scores of the novel EE subscales, while controlling for the other EE subscale scores, age, sex, and education (see Supplementary Table 2, for the correlation matrix of EE and contextual variables; see Supplementary Table 3, for the correlation matrix of the contextual variables). The context variables were grand mean centered to reduce collinearity. The outcome variables as well as the predictors were z-standardized in order to make them directly comparable. The other EE subscale scores were entered in the first block, the demographic control variables followed in the second block, and all context variables were entered in the third block. The data met all assumptions of linear regression analyses (see Supplementary Data 1).

#### Non-parametric tests

We implemented non-parametric statistical tests (Spearman's correlations and Mann-Whitney *U*-tests) in IBM SPSS 21 as the data was ordinal and most variables were not normally distributed (de Winter et al., 2016). As the correlations procedure in SPSS does not provide confidence intervals in the output, the rho!CI macro by Weaver and Koopman (2014) was used. Convergent and discriminant validity of the scores of the novel PAGE-R subscales was assessed by correlating them with positive-, disorganized-, and negative-like symptoms as depicted by the SPQ subscale scores (Raine et al., 1994), STS, SNS, and the PAS. More information about the discriminant validity of the PAGE-R subscale scores was gained by calculating their intercorrelations as well as by correlating them with valence ratings for overall EE, while controlling for the other PAGE-R subscale scores (partial rank correlations). We tested the scores of the novel EE subscales, the positive-like SPQ subscale scores, as well as STS and SNS for significant associations with age, educational achievement, and general psychological burden using Spearman's correlations. The correlational analyses were controlled for multiple comparisons using the false detection rate algorithm (FDR) by Benjamini and Hochberg (1995). We applied *U*-tests and point-biserial correlations to test for significant sex differences regarding EE, PLE, disorganized-, and negative-like symptoms.

#### One sample Z-test

Using a one sample *z*-test we tested whether the mean PAGE-R total score of our sample significantly differed from a sample representative of the general population (Landolt et al., 2014). One sample *z*-tests are used to test the hypothesis that a sample mean is equal to a population mean, when the population standard deviation is known. We applied the *z*-test syntax available on www.how2stats.net.

## Results

### Factor analysis of exceptional experiences (PAGE-R)

An exploratory factor analysis was performed in order to identify latent factors underlying exceptional experiences (EE) in healthy individuals. The analysis indicated two latent factors encompassing odd beliefs and anomalous perceptions. However, the extraction of three factors resulted in two more homogeneous subfactors representing dissociative anomalous perceptions and hallucinatory anomalous perceptions (see Table 1, for the 3-factor solution). Three corresponding scales were created, depicting odd beliefs (OB; α = 0.90), dissociative anomalous perceptions (DAP; α = 0.76), and hallucinatory anomalous perceptions (HAP; α = 0.84). OB correlated moderately with the anomalous perceptions scales (HAP, *r*_*s*_ = 0.63, *p* = 0.000, 95% CI [0.54, 0.71]; DAP, *r*_*s*_ = 0.61, *p* = 0.000, 95% CI [0.52, 0.69]). DAP and HAP also correlated moderately (*r*_*s*_ = 0.60, *p* = 0.000, 95% CI [0.50, 0.68]). OB were most frequently reported, followed by HAP, and DAP (see Table 2, for the descriptive statistics).

**Table 1:** **Factor Loadings for the PAGE-R (Revised Exceptional Experiences Questionnaire) items with a 3-factor solution**.

**Item No**.	**Item**	**F1**	**F2**	**F3**
**ODD BELIEFS**				
20	Anticipation of future events	**0.87**	−0.42	0.13
19	Meaningful coincidences	**0.82**	0.08	−0.14
18	Reading thoughts and feelings	**0.74**	0.07	−0.04
22	Recognition of a hidden order	**0.73**	**0.33**	−0.20
9	Vivid imagination	**0.65**	0.11	−0.10
23	Experiencing past dreams	**0.65**	−0.22	0.26
3	Feeling a presence	**0.65**	0.09	0.14
21	Déjà-vus	**0.62**	−0.15	0.14
10	Strange thoughts	**0.55**	**0.41**	−0.10
17	Spontaneous knowledge of past events	**0.55**	0.17	0.15
7	Inexplicably changing environment	**0.54**	−0.14	**0.35**
**DISSOCIATIVE ANOMALOUS PERCEPTIONS**				
32	External control of the body	−0.19	**1.05**	−0.06
27	Autonomous body activity	−0.20	**0.89**	−0.03
25	Inexplicable bodily alterations	0.08	**0.74**	−0.19
30	Inability to move or speak	−**0.34**	**0.62**	**0.44**
13	Alienations to own personality	0.29	**0.62**	−0.10
14	Inexplicable somatic sensations	0.14	**0.54**	0.16
^*^12	Alienations to own feelings or moods	**0.34**	**0.44**	0.03
16	Manipulated inner experience	0.27	**0.42**	0.23
^*^28	Out of body experiences	**0.36**	**0.38**	0.05
**HALLUCINATORY ANOMALOUS PERCEPTIONS**				
5	Inexplicable noises	0.01	−0.21	**0.94**
4	Hypnagogic perceptions	−0.10	0.08	**0.81**
31	Molestation by invisible agents	−0.22	0.21	**0.76**
15	Encounters during sleep	0.31	−0.27	**0.70**
29	Attacks in hypnagogic states	−0.30	**0.49**	**0.67**
11	Hearing noises or voices in the head	−0.03	0.30	**0.60**
1	Inexplicable visual perceptions	**0.33**	−0.03	**0.55**
2	Autonomously acting objects	−0.06	**0.36**	**0.51**
26	Touches by invisible agents	0.18	0.25	**0.47**
^*^8	Extraordinarily connected events	**0.35**	0.06	**0.40**
^*^24	Occult practices	0.31	0.16	**0.33**

*Factor loadings greater than absolute 0.32 are in boldface (Tabachnick and Fidell, 2012). Item 6 (olfactory sensations) did not load significantly on any of the factors. When using oblique rotations, factor loadings can be greater than one as they are not correlations (Williams and Child, 2003*.

* *Items not included in the corresponding subscales*.

**Table 2:** **Descriptive statistics**.

**Scale**	**Median**	**Mean (*SD*)**	**Range**
Revised Exceptional Experiences Questionnaire (PAGE-R)			
Total score	13	18.13 (16.40)	0–90
Subscale Odd beliefs (OB)	7	9.52 (8.23)	0–36
Subscale Hallucinatory anomalous perceptions (HAP)	2	3.88 (4.50)	0–22
Subscale Dissociative anomalous perceptions (DAP)	1	2.31 (3.02)	0–22
Schizotypal Personality Questionnaire (SPQ)—Total score	17	18.86 (10.56)	0–66
Physical Anhedonia Scale (PAS)	9	10.07 (6.25)	0–30
Revised Symptom Checklist 90 (SCL-90-R)—Global severity index	0.37	0.46 (0.31)	0–1.52

*SD, standard deviation. N = 206*.

#### Analysis of relationship between exceptional experiences subscales and clinical symptoms

In order to assess convergent and discriminant validity, the EE subscale scores were correlated with positive-, disorganized-, and negative-like symptoms (see Table 3, for the correlation matrix). OB, DAP, and HAP correlated with PLE ranging from weak correlations (SPQ suspiciousness, *r*_*s*_ = 0.31) to stronger correlations (SPQ paranormal beliefs, *r*_*s*_ = 0.69). Further, the EE subscale scores correlated weakly with disorganized-like deficits ranging from *r*_*s*_ = 0.21 to *r*_*s*_ = 0.36 (both with SPQ odd speech). In contrast, they correlated in only one instance with negative-like symptoms (SPQ constricted affect, *r*_*s*_ = 0.18).

**Table 3:** **Correlation matrix of exceptional experiences with scales depicting positive-, disorganized-, and negative-like symptoms**.

**Subclinical symptoms**	***r_*s*_* [CI 95%], *p* Exceptional experiences**		
	**Odd beliefs**	**Anomalous perceptions**	
		**Dissociative**	**Hallucinatory**
**POSITIVE-LIKE SYMPTOMS**			
Paranormal beliefs	**0.69 [0.61, 0.76], 0.000**	**0.48 [0.36, 0.57], 0.000**	**0.48 [ 0.37, 0.58], 0.000**
Unusual perceptual experiences	**0.66 [ 0.57, 0.72], 0.000**	**0.54 [0.44, 0.63], 0.000**	**0.55 [0.45, 0.64], 0.000**
Ideas of reference	**0.50 [0.39, 0.59], 0.000**	**0.43 [0.31, 0.53], 0.000**	**0.35 [0.22, 0.46], 0.000**
Suspiciousness	**0.36 [0.23, 0.47], 0.000**	**0.31 [0.18, 0.43], 0.000**	**0.40 [0.28, 0.51], 0.000**
Schizotypal signs	**0.38 [0.25, 0.49], 0.000**	**0.35 [0.23, 0.47], 0.000**	**0.37 [0.25, 0.49], 0.000**
Schizophrenia nuclear symptoms	**0.36 [0.23, 0.47], 0.000**	**0.34 [0.21, 0.45], 0.000**	**0.34 [0.21, 0.45], 0.000**
**DISORGANIZED-LIKE SYMPTOMS**			
Odd speech	**0.36 [0.24, 0.47], 0.000**	**0.21 [0.08, 0.34], 0.002**	**0.34 [0.22, 0.46], 0.000**
Odd behavior	**0.30 [0.17, 0.42], 0.000**	**0.33 [0.21, 0.45], 0.000**	**0.25 [0.12, 0.38], 0.000**
**NEGATIVE-LIKE SYMPTOMS**			
Excessive social anxiety	0.11 [−0.03, 0.24], 0.122	0.10 [−0.04, 0.23], 0.163	*0.14 [0.00, 0.27], 0.044*
Constricted affect	0.11 [−0.03, 0.24], 0.135	*0.13 [0.00, 0.27], 0.057*	**0.18 [0.05, 0.31], 0.009**
No close friends	0.05 [−0.08, 0.19], 0.444	*0.12 [0.01, 0.26], 0.076*	0.06 [−0.08, 0.19], 0.416
Physical Anhedonia Scale	−0.12 [−0.26, 0.02], 0.083	−0.06 [−0.20, 0.08], 0.310	0.00 [−0.14, 0.14], 0.979

*r_s_, Spearman's rho; CI, confidence interval; p, probability. The FDR corrected (Benjamini and Hochberg, 1995) alpha levels were 0.078 (0.10, trend), 0.035 (0.05, significant), and 0.007 (0.01, highly significant). Significant correlations are in boldface, statistical trends in italics*.

#### Analysis of relationship between exceptional experiences subscales and levels of distress/comfort?

The amount of distress and comfort specifically associated with odd beliefs and anomalous perceptions was estimated using partial correlations. Enriching ratings were moderately associated with odd beliefs (OB, *r*_*s*_ = 0.31, *p* = 0.000) and weakly with dissociative anomalous perceptions (DAP, *r*_*s*_ = 0.15, *p* = 0.028). In contrast, hallucinatory anomalous perceptions (HAP) were positively associated with negative valence (*r*_*s*_ = 0.18, *p* = 0.009). Hierarchical multiple linear regressions were conducted to identify specific contexts, in which EE tended to occur (see Table 4, for the regression models). OB, DAP, and HAP were all significantly explained by the scores of the other subscales, the control variables (except HAP), and the contextual variables. OB specifically formed in waking states, in extreme situations, and not against one's own volition. In contrast, DAP occurred against one's own volition. Moreover, DAP specifically occurred during contacts with healers and under the influence of substances. Relatedly, DAP tended to occur when mental techniques were applied. HAP did specifically not occur in waking states and trended toward occurring spontaneously.

**Table 4:** **Hierarchical multiple regression analyses predicting exceptional experiences from contextual variables**.

**Predictor**		**Odd beliefs**	**Anomalous perceptions**	
			**Dissociative**	**Hallucinatory**
Step 1 Other subscales	Δ*R*^2^, *p*	**0.55, 0.000**	**0.50, 0.000**	**0.55, 0.000**
Step 2 Control variables^a^	Δ*R*^2^, *p*	**0.03, 0.010**	**0.03, 0.019**	0.01, 0.146
Step 3 Contextual variables	Δ*R*^2^, *p*	**0.10, 0.000**	**0.08, 0.000**	**0.04, 0.045**
Waking states	β [CI 95%], *p*	**0.17 [0.07, 0.27], 0.001**	−0.06 [−0.17, 0.06], 0.318	−**0.16 [**−**0.27**, −**0.05], 0.006**
Occult practices		0.07 [−0.04, 0.18], 0.221	0.01 [−0.12, 0.13], 0.918	0.06 [−0.06, 0.19], 0.311
Spontaneously		0.08 [−0.02, 0.19], 0.126	−0.08 [−0.19, 0.04], 0.188	*0.11 [−0.01, 0.22], 0.063*
Mental techniques		0.08 [−0.05, 0.20], 0.215	*0.13 [0.00, 0.26], 0.050*	0.09 [0.04, 0.22], 0.170
Contact with healers		−0.06 [− 0.17, 0.04], 0.215	**0.13 [0.02, 0.24], 0.018**	0.04 [−0.07, 0.15], 0.487
Against own volition		−**0.11 [**−**0.21**, −**0.02], 0.018**	**0.19 [0.09, 0.29], 0.000**	0.03 [−0.07, 0.14], 0.525
Drug-induced		−0.04 [−0.13, 0.05], 0.351	**0.11 [0.02, 0.21], 0.023**	−0.02 [−0.12, 0.08], 0.729
On own volition		0.05 [−0.07, 0.18], 0.385	0.04 [−0.09, 0.17], 0.560	0.06 [−0.07, 0.20], 0.355
Extreme situations		**0.14 [0.05, 0.23], 0.002**	0.00 [−0.10, 0.10], 0.974	−0.08 [−0.18, 0.02], 0.100
Total *R^2^*		0.67	0.60	0.60

a *Control variables include age, sex, and educational achievement. R^2^, explained variance; p, probability; β, standardized regression weight; CI, confidence interval. The alpha levels were 0.10 (trend), 0.05 (significant), and 0.01 (highly significant). Significant results are in boldface, statistical trends in italics*.

### Associations of exceptional experiences and positive-like symptoms with age, educational achievement, and psychological burden

Scores of odd beliefs (OB), dissociative anomalous perceptions (DAP), hallucinatory anomalous perceptions (HAP), the positive-like SPQ subscales, schizotypal signs (STS), and schizophrenia nuclear symptoms (SNS), were tested for significant associations with age, educational achievement, and general psychological burden (GSI; see Table 5, for the correlation matrix). Age correlated positively with paranormal beliefs and trended toward correlating negatively with STS. Educational achievement was negatively associated with OB, HAP, paranormal beliefs, unusual perceptual experiences, and suspiciousness. The GSI positively correlated with OB, DAP, HAP, ideas of reference, paranormal beliefs, unusual perceptual experiences, suspiciousness, STS, and SNS. In summary, we consistently found that PLE and EE correlated positively with general psychological burden and negatively with higher educational achievement.

**Table 5:** **Correlation matrix of EE and positive-like symptoms with age, educational achievement, and psychological burden**.

**Positive-like symptoms**	***r_*s*_* [CI 95%], *p***		
	**Age**	**Educational achievement**	**General psychological burden (GSI)**
**PAGE-R**			
Odd beliefs	0.07 [−0.07, 0.21], 0.319	**−0.27 [−0.39, −0.14], 0.000**	**0.41 [0.29, 0.52], 0.000**
Dissociative anomalous perceptions	0.02 [−0.12, 0.16], 0.758	−0.07 [−0.20, 0.07], 0.321	**0.42 [0.30, 0.52], 0.000**
Hallucinatory anomalous perceptions	0.02 [−0.12, 0.15], 0.825	**−0.30 [−0.42, −0.17], 0.000**	**0.46 [0.34, 0.56], 0.000**
**SPQ**			
Paranormal beliefs	**0.31 [0.18, 0.42], 0.000**	**−0.19 [−0.31, −0.05], 0.008**	**0.28 [0.15, 0.40], 0.000**
Unusual perceptual experiences	0.01 [−0.13, 0.14], 0.938	**−0.19 [−0.32, −0.05], 0.007**	**0.45 [0.33, 0.55], 0.000**
Ideas of reference	−0.10 [−0.23, 0.04], 0.157	0.02 [−0.12, 0.16], 0.779	**0.46 [0.35, 0.57], 0.000**
Suspiciousness	−0.01 [−0.15, 0.13], 0.903	**−0.16 [−0.29, −0.02], 0.024**	**0.42 [0.30, 0.53], 0.000**
**SCL-90-R**			
Schizotypal signs	*−0.14 [−0.27, 0.00], 0.048*	−0.11 [−0.24, 0.03], 0.118	**0.73 [0.66, 0.79], 0.000**
Schizophrenia nuclear symptoms	−0.07 [−0.20, 0.07], 0.355	−0.07 [−0.21, 0.06], 0.297	**0.42 [0.30, 0.53], 0.000**

*r_s_, Spearman's rho; CI, confidence interval; p, probability. The FDR corrected (Benjamini and Hochberg, 1995) alpha levels were 0.052 (0.10, trend), 0.028 (0.05, significant), and 0.004 (0.01, highly significant). PAGE-R, Revised Exceptional Experiences Questionnaire; SPQ, Schizotypal Personality Questionnaire; SCL-90-R, Revised Symptom Checklist 90. Significant correlations are in boldface, statistical trends in italics*.

### Gender analysis of exceptional experiences and subclinical symptoms

Scores of odd beliefs (OB), dissociative anomalous perceptions (DAP), hallucinatory anomalous perceptions (HAP), the SPQ subscales, schizotypal signs (STS), schizophrenia nuclear symptoms (SNS), and the Physical Anhedonia Scale (PAS) were tested for sex differences. Women (*Mdn* = 9) scored higher than men (*Mdn* = 6) on OB (*U* = 3937, *Z* = −2.28, *p* = 0.023, *r* = 0.16, 95% CI [0.02, 0.29]). Women also trended toward reporting more HAP (*Mdn* = 3) than men (*Mdn* = 2; *U* = 3825, *Z* = −1.18, *p* = 0.069, *r* = 0.13, 95% CI [0.01, 0.26]). Further, women scored higher than men on paranormal beliefs (*Mdn* = 3, *Mdn* = 1, *U* = 3539, *Z* = −3.27, *p* = 0.001, *r* = 0.23, 95% CI [0.10, 0.35]) and excessive social anxiety (*Mdn* = 2, *Mdn* = 1, *U* = 3145, *Z* = −4.28, *p* = 0.000, *r* = 0.30, 95% CI [0.17, 0.42]). Men reported more symptoms of odd behavior (*Mdn* = 1) than women (*Mdn* = 0, *U* = 4076, *Z* = −1.98, *p* = 0.048, *r* = 0.14, 95% CI [0.00, 0.27]) and they trended toward a higher mean score on physical anhedonia (*Mdn* = 9.5) than women (*Mdn* = 9, *U* = 3978, *Z* = −1.91, *p* = 0.056, *r* = 0.14, 95% CI [0.00, 0.27]). No significant sex differences were found regarding STS or SNS scores. In summary, we found that women scored higher on positive-like symptoms and EE but lower on disorganized-like symptoms than men.

### Mean reported exceptional experiences in comparison with the general population

Using a one sample *z*-test we compared the present sample to a general population sample regarding the mean level of reported overall EE (PAGE-R total score; Landolt et al., 2014). Our sample reported slightly more EE (*M* = 20.36, *SD* = 17.63) than a sample representative of the general population (*M* = 17.43, *SD* = 18.14; *Z* = 2.32, *p* = .020, *d* = 0.16).

## Discussion

In order to untangle the complex architecture of psychotic disorder, the continuum from health to disease needs to be accurately mapped out. Here we explored PLE at the healthy end of the psychosis continuum and incorporated the PAGE-R questionnaire inquiring exceptional experiences. Exploratory factor analyses and correlational cross validation of EE supported the initial hypothesis that the PAGE-R captures facets of PLE. In line with a continuous distribution of psychotic experiences and associated factors in the general population, EE and PLE in healthy individuals were indicative of reduced psychological functioning. Moreover, we detected similar sex differences as in clinical and general population samples. However, odd beliefs were particularly enriching and might therefore also be implicated in the maintenance of psychological functioning to some degree. We suggest that the PAGE-R might be useful for a more comprehensive description of PLE.

### Psychotic-like exceptional experiences

In order to assess convergent and discriminant validity of the PAGE-R, the subscale scores were correlated with subscale scores for positive-, disorganized-, and negative-like symptoms. The results indicated a distinct overlap between EE and positive-like symptoms. Notably, the correlational overlap between scales assessing EE and PLE extended to most single EE as well (see Supplementary Tables 4, 5). In contrast, EE subscale scores were less strongly associated with scores of disorganized-like deficits and only partially and weakly with negative-like symptom scores. Notably, in the present sample, EE subscale scores were similarly related to disorganized- and negative-like symptom scores as were the scores measuring positive-like symptoms (see Supplementary Table 6, for the correlation matrix), except for SPQ suspiciousness and schizotypal signs (STS), which are conceptually related to some negative-like symptoms. Moreover, similar correlational patterns between scores for positive-, disorganized-, and negative-like symptoms have been found in other studies using the SPQ (see Raine et al., 1994). Earlier findings of significant correlations between scores of positive- and disorganized-like symptoms have led to suggestions that the positive and disorganized SPQ factors might not be distinct dimensions (Gross et al., 2014). Furthermore, both dimensions might be closely related or indistinguishable in schizophrenia as well (Andreasen et al., 1990; Fenton and McGlashan, 1991; Brekke et al., 1994). The initial exploratory factor analysis indicated that the two higher-order factors odd beliefs and anomalous perceptions underlie exceptional experiences in healthy individuals. This finding is similar to previous studies (Rössler et al., 2007, 2013, 2015) that demonstrated that sub-clinical psychosis can be reduced to the two broad categories of odd beliefs/behaviors and anomalous perceptions that parallel the delusions and hallucinations of full-blown psychosis. Furthermore, in accordance with the studies of Rössler and others (see van Os et al., 2009), the present population reported more odd beliefs than anomalous perceptions. The present results support our initial hypothesis that EE reflect aspects of positive-like symptoms. Importantly, the two-dimensional model of EE might allow directly relating EE with other psychotic experiences across the psychosis continuum. However, testing for equivalence of measures (or measurement invariance) across healthy and psychotic samples e.g., using confirmatory factor analysis would be required to establish construct similarity between EE and psychotic(-like) experiences (Milfont and Fischer, 2010).

### Valence and context of occurrence of exceptional experiences

Three EE subscales corresponding to odd beliefs, dissociative anomalous perceptions, and hallucinatory anomalous perceptions were analyzed regarding specific associations with valence and context of occurrence. The differential and meaningful associations of the PAGE-R subscale scores with valence ratings and context of occurrence supported the validity of the three EE factors odd beliefs, dissociative anomalous perceptions, and hallucinatory anomalous perceptions underlying EE in healthy individuals. Odd beliefs were foremost characterized by the meaningful linking of separate events, and further, the over interpretation of mental states, vivid imagination, and felt presences. Notably, a felt presence does not have a sensory component in a strict sense, which is why it has been classified as a delusional-like experience (Cheyne, 2012). Odd beliefs were particularly associated with positive/enriching ratings. Regression modeling suggested that odd beliefs specifically tended to form in waking states, not against one's own volition, and in extreme situations. This might reflect that odd beliefs arose in situations where revelations, answers or help were actively sought. This is consistent with suggestions that such experiences might be a means to reduce distress in perceptually ambiguous or stressful situations (Beitman, 2009), re-establish (perceived) control under lack of control, and create confidence and agency (Whitson and Galinsky, 2008). Hence, despite their delusion-like quality, odd beliefs hold the potential to exert a positive influence on psychological functioning to some degree. As research has often focused on negatively-valenced experiences, conclusions about the significance of PLE for mental health might have been biased (Wiseman and Watt, 2004; Perdue, 2013). In that respect, the PAGE-R odd beliefs subscale might be useful for the assessment of positive PLE in the study of subclinical psychosis.

Anomalous perceptions were characterized by bodily dissociations and hallucinatory experiences. Interestingly, dissociative anomalous perceptions were associated with positive ratings although it has been found that PLE with lack of control are particularly distressing (Bak et al., 2003). This might indicate that healthy individuals accept or even welcome dissociative anomalous perceptions when they occur as side effects of mental techniques or the use of substances, as the regression analyses suggested. The tendency of dissociative anomalous perceptions to be associated with the use of mental techniques might be explained by the fact that body dissociation is intended in certain meditation techniques (del C Quezada-Berumen et al., 2014). Hallucinatory anomalous perceptions were particularly distressing, occurred not during wakefulness and tended to occur surprisingly. This fits well with the observation that many of these experiences either explicitly or typically occur in sleep-related states, such as sleep paralysis (Cheyne and Girard, 2007).

A recent meta-analysis of factor analytic studies on the Community Assessment of Psychic Experience (CAPE; Stefanis et al., 2002) suggested that 3 factors also underlie its 20 positive-like symptoms (Mark and Toulopoulou, 2016). Interestingly, these dimensions parallel the PAGE-R dimensions odd beliefs (*cf*. delusional ideations), dissociative anomalous perceptions (*cf*. bizarre experiences), and hallucinatory anomalous perceptions (*cf*. perceptual anomalies). Although the CAPE and the PAGE-R are clearly separable instruments, the similarities in factor structure and factor content might further support the validity of a tripartite factor structure underlying the PAGE-R. Odd beliefs, dissociative anomalous perceptions, and hallucinatory anomalous perceptions might extend commonly studied forms of PLE as they are likely to capture subtler and partly neglected forms of experiences. For example, the particular positive valence of odd beliefs contrasts with other forms of delusion-like beliefs such as paranoid ideation that are usually studied in research on subclinical psychosis. Similarly, hallucinatory anomalous perceptions explicitly include experiences in hypnagogic states, which are often not considered in research on (subclinical) psychosis (e.g., van Os et al., 2009). The close association of odd beliefs, dissociative anomalous perceptions, and hallucinatory anomalous perceptions might support the notion that dissociations, sleep experiences and positive-like symptoms form part of one common domain of imaginative abilities (Watson, 2001, 2003; Fassler et al., 2006; Knox and Lynn, 2014). Importantly, these experiences are not only thought to manifest in waking states but across different states of consciousness. As our data indicate, these states might include extreme situations, sleep-related states, drug-induced states or meditation.

### Evidence for similar sex differences across the psychosis continuum

Our results tie in with studies that found sex differences in PLE and associated disorganized- and negative-like symptoms. More specifically, we could replicate that women tend to obtain higher scores on PLE and EE but lower scores on disorganized- and negative-like symptoms (trend) than men (Raine, 1992; Rawlings et al., 2001; Fossati et al., 2003; Maric et al., 2003; Mata et al., 2005; Bora and Baysan Arabaci, 2009). Importantly, studies indicate that female patients suffering from schizophrenia may exhibit more positive and affective symptoms than men and less severe disorganized and negative symptoms as well (Goldstein et al., 1990; Rector and Seeman, 1992; Shtasel et al., 1992; Gur et al., 1996; Schultz et al., 1997; Roy et al., 2001; Ochoa et al., 2012). In a large longitudinal study incorporating a community cohort, Rössler et al. (2012) investigated sex differences in symptoms related to full-blown psychosis as depicted by schizotypal signs (STS) and schizophrenia nuclear symptoms (SNS). As no sex differences regarding these symptoms were found, the authors concluded that sex differences rather express themselves at clinical than subclinical levels. Interestingly, whereas we attained similar results as other studies using the SPQ and the PAGE-R, we did not find sex differences with respect to the scores of STS or SNS either. A number of factors such as sampling biases might explain why some studies did find sex differences in subclinical psychosis and others did not. However, our result might indicate that sex differences do exist in subclinical samples, but can more easily be detected when subtler or clinically less relevant symptoms are assessed than STS or SNS. Notably, as we implemented more scales assessing positive-like than disorganized-, or negative-like symptoms, the chances to find sex differences regarding PLE was increased. Hence, the present results are in need of replication and more data covering different forms of positive-, disorganized-, and negative-like symptoms are needed.

### Psychotic-like experiences in healthy individuals might differentially affect functioning

The correlational analyses revealed that also in healthy individuals, most EE and PLE were indicative of reduced functioning: They were associated with distress, lower educational achievement, general psychological burden, as well as disorganized- and negative-like symptoms. Importantly, these results tie in with earlier results in clinical and subclinical samples (see Linscott and van Os, 2013) and match what we would expect to find at the healthy end of the psychosis continuum if the continuum hypothesis held true. Surprisingly, the frequency of paranormal beliefs (SPQ) increased with age. However, this association might be explained by the relatively high proportion of “have you had” questions in this scale that contrasts with the great majority of items in the SPQ assessing the *current* frequency of experiences (see Raine, 1991).

It is possible that the negative associations between PLE and educational achievement reflect that PLE negatively affect normal functioning, which is notably a general criterion for mental disorders in DSM and ICD. However, the cross-sectional data do not allow inferring any causal relations and educational achievement is closely related to social and socio-economic status as well. Therefore, it is also possible that stress associated with social isolation is causal for both, lower educational achievement and PLE (Scott et al., 2008). Regarding EE, hallucinatory anomalous perceptions were particularly distressing and were therefore the most likely type of EE to entail a need of care or treatment (Murphy et al., 2012). Although lack of control has been shown to characterize distressing PLE (Bak et al., 2003), dissociative anomalous perceptions were rather endorsed positively. We suggested that the context of their occurrence in healthy individuals (e.g., meditation) might facilitate their positive appraisal (see Section Valence and Context of Occurrence of Exceptional Experiences). Fittingly, Peters et al. (2016) have shown that individuals with persistent PLE but without need for care lack paranoid appraisals of their experiences. Therefore, the presence of psychological mechanisms that allow the positive appraisal of PLE might be pivotal for the maintenance of mental health despite the presence of PLE. As stated above, the particularly enriching formation of odd beliefs might offer such a means to successfully cope with burdensome or unusual experiences such as hallucinatory anomalous perceptions. When not corrected for multiple comparisons, odd beliefs trended toward a *negative* association with the PAS. Relatedly, SPQ paranormal beliefs were negatively associated with physical anhedonia (see Supplementary Table 6). These results are similar to other “paradoxical” findings showing negative associations between paranormal beliefs and the PAS (Chapman et al., 1982) or depression (Yung et al., 2006). These observations might support the notion that odd or paranormal beliefs arise as cognitive responses to distressing experiences and help to maintain mental functioning (e.g., Whitson and Galinsky, 2008). Hence, whereas PLE seem to be indicative of reduced psychological functioning, some of them might somewhat paradoxically also be involved in the maintenance of mental functioning. The identification of such psychological coping mechanisms might advance our understanding of resilience to psychosis and could be exploited for therapeutic psychosis prevention. Future studies need to replicate the present results and to investigate the interplay of different PLE and associated symptoms in the dynamics of psychosis formation.

### Limitations

The present findings need to be interpreted in the light of several limitations. The external validity of the results is limited as this research was confined to predominantly young and well-educated participants. Furthermore, diagnoses and family history of mental disorders were assessed by self-report and were not verified through diagnostic interviews. Also the assessment of “caseness” is not a state-of-the-art methodology in capturing mental health states, it rather represents an unspecific pathology as it appears in the beginnings of psychiatric disorders (Mueller et al., 2009). Thus, one issue for future research would be to incorporate diagnostic interviews to control for psychopathology. Due to the multi-methodological approach of this research project toward studying PLE in healthy individuals the number of subjects was limited. Although the present sample size may be sufficient for performing exploratory factor analyses (Guilford, 1954; Gorsuch, 1974), larger sample sizes are desired in future analyses in order to increase the reliability of the results (Tabachnick and Fidell, 2012). In the present study, we calculated subscale scores on the basis of a three-factor solution. However, this decision might have come at the cost of model parsimony and discriminant validity of the resulting subscale scores. Further examinations are required to determine whether using subscales on the basis of a two- or three-factor solution of the PAGE-R proves to be more useful. The present sample reported slightly more overall EE (PAGE-R total score) than a general population sample. As this sample might have been biased toward individuals with more and perhaps positively-valenced EE, the analyses need replication across other samples, e.g., individuals at high risk for psychosis. Relatedly, we cannot exclude that some participants also reported hypnagogic experiences as PLE, which might have influenced the correlations between EE and PLE. Therefore, this aspect should be controlled for in future studies as well. Due to the cross-sectional design of this study, the inferences about the psychopathological significance of EE are limited. A longitudinal study design may help to disentangle which experiences represent risk factors or not.

## Conclusion

Our results indicate that the association of psychotic experiences with socio-demographic factors, comorbid symptoms, and psychological distress might extend to healthy individuals from the general population. At the same time, our results support the notion that subtypes of PLE might differentially be implicated in mental health and well-being (Yung and Lin, 2016). Particularly, the significance of positively-valenced odd beliefs and paranormal beliefs for mental health requires further investigation. We hypothesize that they have a psychologically stabilizing function and form in response to burdensome experiences. A longitudinal study design might help to clarify which PLE indicate a higher risk for transition to psychosis or might serve as protective factors, respectively. The implementation of the PAGE-R in research on subclinical psychosis might be useful for a more comprehensive assessment of PLE, as it might capture three attenuated types of PLE that have often been neglected in research on (subclinical) psychosis. Whereas, our results might point toward a continuity of psychotic symptoms with EE and normal experiences (van Os et al., 2009), they require replication in larger samples as well as equivalence testing across the psychosis continuum, i.e. from healthy to clinical population samples.

## Author contributions

LU contributed to the acquisition of data, analyzed, and interpreted the data and drafted the manuscript. TW contributed to the acquisition of data and critically revised the manuscript. DW, and VA contributed to the interpretation of the data and revised the manuscript critically. HH, and WR contributed to the conception and design of the study and revised the manuscript critically. All authors have given final approval for the version to be published and agreed to be accountable for all aspects of the work.

## Funding

This research was supported by the Zurich Program for Sustainable Development of Mental Health Services (ZInEP) and the Dr. Donald C. Cooper Fond of the ETH Zurich.

### Conflict of interest statement

The authors declare that the research was conducted in the absence of any commercial or financial relationships that could be construed as a potential conflict of interest. The reviewer JK and the handling Editor declared their shared affiliation, and the handling Editor states that the process nevertheless met the standards of a fair and objective review.
