# Supplementary material for: Psychotic-Like Experiences at the Healthy End of the Psychosis Continuum
Source: Front Psychol. 2017 May 15;8:775. doi: 10.3389/fpsyg.2017.00775 (PMC5431212; doi:10.3389/fpsyg.2017.00775)
Supplement: Supplementary file 1 [file Table1.docx]

Supplementary Material

Psychotic-Like Experiences at the Healthy End of the Psychosis Continuum

Lui Unterrassner^1^*, Thomas Wyss^1^, Diana Wotruba^1^, Vladeta Ajdacic-Gross^2^, Helene Haker^1,3^, and Wulf Rössler^1,2,4^

*** Correspondence:** Corresponding Author: unterrassner@collegium.ethz.ch

**Supplementary Table 1**

**Factor Loadings for the PAGE-R Items with a 2-Factor Solution.** Factor loadings greater than absolute .32 are in boldface (Tabachnick & Fidell, 2012). When using oblique rotations, factor loadings can be greater than one as they are not correlations (Williams & Child, 2003).

| Item No. | Item | F1 | F2 |
| --- | --- | --- | --- |
|  |  |  |  |
| Odd Beliefs | |  |  |
| 20 | Anticipation of future events | **1.04** | **-0.33** |
| 19 | Meaningful coincidences | **0.92** | -0.21 |
| 18 | Reading thoughts and feelings | **0.85** | -0.12 |
| 23 | Experiencing past dreams | **0.81** | -0.17 |
| 22 | Recognition of a hidden order | **0.79** | 0.01 |
| 3 | Feeling a presence | **0.77** | 0.05 |
| 21 | Déjà-vus | **0.75** | -0.18 |
| 9 | Vivid imagination | **0.73** | -0.10 |
| 7 | Inexplicably changing environment | **0.70** | 0.00 |
| 17 | Spontaneous knowledge of past events | **0.66** | 0.15 |
| 10 | Strange thoughts | **0.60** | 0.21 |
| 15 | Encounters during sleep | **0.52** | 0.19 |
| 1 | Inexplicable visual perceptions | **0.50** | 0.30 |
| 8 | Extraordinarily connected events | **0.48** | 0.28 |
| 24 | Occult practices | **0.41** | **0.33** |
| 28 | Out of body experiences | **0.41** | **0.33** |
| 12 | Strange feelings | **0.38** | **0.38** |
|  |  |  |  |
| Anomalous Perceptions | |  |  |
| 29 | Attacks in hypnagogic states | -0.25 | **1.04** |
| 32 | External control of the body | -0.29 | **1.03** |
| 30 | Inability to move or speak | **-0.34** | **1.01** |
| 27 | Autonomous body activity | -0.28 | **0.89** |
| 31 | Molestation by invisible agents | -0.12 | **0.81** |
| 2 | Autonomously acting objects | 0.02 | **0.74** |
| 11 | Strange noises or voices in the head | 0.06 | **0.74** |
| 4 | Hypnagogic perceptions | 0.05 | **0.70** |
| 14 | Inexplicable somatic sensations | 0.17 | **0.61** |
| 25 | Inexplicable bodily alterations | 0.02 | **0.57** |
| 26 | Touches by invisible agents | 0.29 | **0.56** |
| 16 | Manipulated inner experience | **0.34** | **0.53** |
| 5 | Inexplicable noises | 0.20 | **0.48** |
| 13 | Alienation to own personality | 0.28 | **0.47** |
| 6 | Olfactory sensations | 0.27 | 0.29 |

**References**

Tabachnick, B. G., & Fidell, L. S. (2012). *Using multivariate statistics*. *New York: Harper and Row* (6th ed.). Boston: Pearson.

Williams, J. S., & Child, D. (2003). *The Essentials of Factor Analysis.* *Contemporary Sociology* (Vol. viii). http://doi.org/10.2307/2061984
