# Supplementary material for: Psychotic-Like Experiences at the Healthy End of the Psychosis Continuum
Source: Front Psychol. 2017 May 15;8:775. doi: 10.3389/fpsyg.2017.00775 (PMC5431212; doi:10.3389/fpsyg.2017.00775)
Supplement: Supplementary file 2 [file Table2.docx]

Supplementary Material

Psychotic-Like Experiences at the Healthy End of the Psychosis Continuum

Lui Unterrassner^1^*, Thomas Wyss^1^, Diana Wotruba^1^, Vladeta Ajdacic-Gross^2^, Helene Haker^1,3^, and Wulf Rössler^1,2,4^

*** Correspondence:** Corresponding Author: unterrassner@collegium.ethz.ch

**Supplementary Table 2**

**Correlation Matrix of Exceptional Experiences and Contextual Variables.** *r_s_* = Spearman’s rho; CI = confidence interval. The FDR corrected (Benjamini & Hochberg, 1995) alpha levels were .085 (.10, *trend*), .037 (.05, **significant**), and .007 (.01, **highly** **significant**).

|  |  |  |  |  |  | |
| --- | --- | --- | --- | --- | --- | --- |
|  |  | *r_s_* [CI 95%], *p* | | | | |
|  |  |  |  |  |  | |
|  |  | Odd beliefs |  | Anomalous Perceptions | | |
|  |  |  |  | Dissociative |  | Hallucinatory |
| Wakefulness |  | **.33 [.20, .45], .000** |  | **.21 [.08, .34], .002** |  | *.13 [-.01, .26], .066* |
| Occult practices |  | **.35 [.22, .46], .000** |  | **.37 [.25, .48], .000** |  | **.30 [.17, .42], .000** |
| Surprisingly |  | .07 [-.07, .21], .298 |  | -.09 [-.23, .05], .193 |  | .05 [-.09, .19], .471 |
| Mental techniques |  | **.44 [.32, .54], .000** |  | **.45 [.34, .56], .000** |  | **.40 [.28, .51], .000** |
| Contact with healers |  | **.25 [.11, .37], .000** |  | **.31 [.19, .43], .000** |  | **.30 [.17, .42], .000** |
| Against own volition |  | .01 [-.13, .14], .974 |  | *.06 [-.07, .20], .053* |  | *.12 [-.02, .25], .083* |
| Drug-induced |  | **.20 [.06, .32], .005** |  | **.32 [.19, .43], .000** |  | **.18 [.04, .31], .010** |
| On own volition |  | **.34 [.22, .46], .000** |  | **.40 [.28, .51], .000** |  | **.29 [.16, .41], .000** |
| Extreme situations |  | **.34 [.21, .45], .000** |  | **.27 [.14, .39], .000** |  | **.25 [.12, .38], .000** |

**References**

Benjamini, Y., & Hochberg, Y. (1995). Controlling the False Discovery Rate: A Practical and Powerful Approach to Multiple Testing. *Journal of the Royal Statistical Society. Series B (Methodological)*, *57*, 289–300. http://doi.org/10.2307/2346101
