# Supplementary material for: Psychotic-Like Experiences at the Healthy End of the Psychosis Continuum
Source: Front Psychol. 2017 May 15;8:775. doi: 10.3389/fpsyg.2017.00775 (PMC5431212; doi:10.3389/fpsyg.2017.00775)
Supplement: Supplementary file 3 [file Table3.docx]

Supplementary Material

Psychotic-Like Experiences at the Healthy End of the Psychosis Continuum

Lui Unterrassner^1^*, Thomas Wyss^1^, Diana Wotruba^1^, Vladeta Ajdacic-Gross^2^, Helene Haker^1,3^, and Wulf Rössler^1,2,4^

*** Correspondence:** Corresponding Author: unterrassner@collegium.ethz.ch

**Supplementary Table 3**

**Correlation Matrix of Contextual Variables.** *r_s_* = Spearman’s rho; CI = confidence interval. The FDR corrected (Benjamini & Hochberg, 1995) alpha levels were .066 (.10, *trend*), .029 (.05, **significant**), and .004 (.01, **highly** **significant**).

|  |  |  |  |  |  |  |  |  |
| --- | --- | --- | --- | --- | --- | --- | --- | --- |
|  |  | *r_s_*, [CI 95%], *p* | | | | | | |
|  |  |  |  |  |  |  |  |  |
|  |  | Wakefulness |  | Occult practices |  | Surprisingly |  | Mental techniques |
| Occult practices |  | .**23 [.09, .35], .001** |  |  |  |  |  |  |
| Surprisingly |  | **.27 [.14, .39], .000** |  | **-.23 [-.36, -.10], .001** |  |  |  |  |
| Mental techniques |  | **.21 [.08, .34], .002** |  | **.40 [.28, .51], .000** |  | **-.18 [-.31, -.05], .009** |  |  |
| Contact with healers |  | **.27 [.14, .39], .000** |  | **.46 [.35, .56], .000** |  | **-.19 [-.32, -.05], .006** |  | **.39 [.26, .50], .000** |
| Against own volition |  | .04 [-.09, .18], .541 |  | -.11 [-.24, .03], .125 |  | **.37 [.25, .48], .000** |  | *-.14 [-.27, .00], .051* |
| Drug-induced |  | **.15 [.02, .28], .028** |  | .09 [-.04, .23], .181 |  | -.08 [-.21, .06], .284 |  | **.27 [.13, .39], .000** |
| On own volition |  | **.30 [.17, .42], .000** |  | **.53 [.43, .63], .000** |  | **-.30 [-.42, -.17], .000** |  | **.54 [.44, .63], .000** |
| Extreme situations |  | .06 [-.08, .20], .377 |  | .12 [-.02, .25], .093 |  | .04 [-.10, .17], .589 |  | *.14 [.01, .27], .041* |
|  |  |  |  |  |  |  |  |  |
|  |  | Contact with healers |  | Against own volition |  | Drug-induced |  | On own volition |
| Against own volition |  | *-.15 [-.28, -.01], .035* |  |  |  |  |  |  |
| Drug-induced |  | **.17 [.03, .30], .015** |  | .00 [-.13, .14], .943 |  |  |  |  |
| On own volition |  | **.38 [.26, 49], .000** |  | **-.15 [-.29, -.02], .024** |  | **.30 [.17, .42], .000** |  |  |
| Extreme situations |  | **.02 [-.12, .16], .758** |  | **.09 [-.05, .22], .217** |  | .12 [-.02, .25], .099 |  | .07 [-.06, .21], .296 |

**References**

Benjamini, Y., & Hochberg, Y. (1995). Controlling the False Discovery Rate: A Practical and Powerful Approach to Multiple Testing. *Journal of the Royal Statistical Society. Series B (Methodological)*, *57*, 289–300. http://doi.org/10.2307/2346101
