# Supplementary material for: Psychotic-Like Experiences at the Healthy End of the Psychosis Continuum
Source: Front Psychol. 2017 May 15;8:775. doi: 10.3389/fpsyg.2017.00775 (PMC5431212; doi:10.3389/fpsyg.2017.00775)
Supplement: Supplementary file 4 [file Table4.DOCX]

Supplementary Material

Psychotic-Like Experiences at the Healthy End of the Psychosis Continuum

Lui Unterrassner^1^*, Thomas Wyss^1^, Diana Wotruba^1^, Vladeta Ajdacic-Gross^2^, Helene Haker^1,3^, and Wulf Rössler^1,2,4^

*** Correspondence:** Corresponding Author: unterrassner@collegium.ethz.ch

**Supplementary Table 4**

**Correlation Matrix of Exceptional Experiences and PLE (First Part).** See Appendix 8, for the second half of the correlation matrix. *r_s_* = Spearman’s rho; CI = confidence interval. The FDR corrected (Benjamini & Hochberg, 1995) alpha levels were .094 (.10, *trend*), .046 (.05, **significant**), and .008 (.01, **highly** **significant**).

|  |  |  |  |  |  |  |
| --- | --- | --- | --- | --- | --- | --- |
|  |  | *r_s_* [CI 95%], *p* | | | | |
|  |  |  |  |  |  |  |
| Item No. |  | SCL-90-R Schizophrenia nuclear symptoms |  | SCL-90-R  Schizotypal signs |  | SPQ  Paranormal beliefs |
| 01 |  | **.305 [.176, .424], .000** |  | **.274 [.143, .396], .000** |  | **.481 [.368, .579], .000** |
| 02 |  | **.306 [.177, .425], .000** |  | **.323 [.195, .441], .000** |  | **.310 [.181, .429], .000** |
| 03 |  | **.245 [.112, .369], .000** |  | **.236 [.102, .361], .001** |  | **.613 [.520, .691], .000** |
| 04 |  | **.294 [.164, .414], .000** |  | **.271 [.139, .393], .000** |  | **.326 [.199, .443], .000** |
| 05 |  | **.288 [.158, .409], 000** |  | **.350 [.224, .465], .000** |  | **.270 [.138, .392], .000** |
| 06 |  | **.331 [.204, .448], 000** |  | **.213 [.078, .340], .002** |  | **.169 [.033, .299], .015** |
| 07 |  | **.184 [.049, .313], .008** |  | **.213 [.079, .340], .002** |  | **.453 [.338, .556], .000** |
| 08 |  | **.149 [.013, .280], .032** |  | *.136 [.000, .268], .051* |  | **.336 [.208, .452], .000** |
| 09 |  | **.178 [.042, .307], .011** |  | **.208 [.073, .335], .003** |  | **.354 [.229, .468], .000** |
| 10 |  | **.418 [.299, .525], .000** |  | **.278 [.146, .399], .000** |  | **.472 [.359, .572], .000** |
| 11 |  | **.405 [.284, .513], .000** |  | **.191 [.056, .320], .006** |  | **.237 [.104, .362], .001** |
| 12 |  | **.351 [.225, .466], .000** |  | **.284 [.153, .405], .000** |  | **.408 [.287, .516], .000** |
| 13 |  | **.180 [.045, .310], .009** |  | **.241 [.108, .366], .000** |  | **.308 [.179, .427], .000** |
| 14 |  | **.260 [.128, .383], .000** |  | **.288 [.157, .408], .000** |  | **.436 [.318, .540], .000** |
| 15 |  | **.190 [.055, .319], .006** |  | **.206 [.072, .334], .003** |  | **.329 [.202, .446], .000** |
| 16 |  | **.241 [.108, .365], .000** |  | **.270 [.138, .392], .000** |  | **.502 [.393, .598], .000** |
| 17 |  | **.225 [.091, .351], .001** |  | **.191 [.056, .319], .006** |  | **.503 [.394, .599], .000** |
| 18 |  | **.205 [.070, .332], .003** |  | **.260 [.128, .383], .000** |  | **.544 [.440, .633], .000** |
| 19 |  | **.320 [.191, .437], .000** |  | **.342 [.216, .458], .000** |  | **.533 [.428, .624], .000** |
| 20 |  | **.247 [.114, .371], .000** |  | **.279 [.148, .400], .000** |  | **.521 [.414, .614], .000** |
| 21 |  | **.216 [.081, .342], .002** |  | **.248 [.115, .372], .000** |  | **.374 [.250, .486], .000** |
| 22 |  | **.358 [.233, .472], .000** |  | **.307 [.178, .426], .000** |  | **.559 [.457, .646], .000** |
| 23 |  | **.257 [.124, .380], .000** |  | **.281 [.150, .402], .000** |  | **.403 [.282, .511], .000** |
| 24 |  | **.181 [.045, .310], .009** |  | .112 [-.025, .245], .110 |  | **.575 [.476, .660], .000** |
| 25 |  | **.281 [.151, .403], .000** |  | **.248 [.115, .372], .000** |  | .114 [-.023, .247], .103 |
| 26 |  | **.266 [.135, .389], .000** |  | **.207 [.072, .334], .003** |  | **.485 [.373, .583], .000** |
| 27 |  | **.175 [.039, .305], .012** |  | **.167 [.031, .297], .016** |  | **.147 [.010, .278], .035** |
| 28 |  | .115 [-.022, .248], .100 |  | **.210 [.076, .337], .002** |  | **.480 [.367, .578], .000** |
| 29 |  | **.239 [.106, .364], .001** |  | **.217 [.083, .344], .002** |  | **.210 [.075, .337], .003** |
| 30 |  | **.200 [.066, .328], .004** |  | **.187 [.052, .316], .007** |  | *.139 [.002, .270], .047* |
| 31 |  | **.153 [.017, .284], .028** |  | .011 [-.126, .148], .872 |  | *.123 [-.014, .255], .079* |
| 32 |  | **.290 [.160, .411], .000** |  | **.162 [.026, .292], .020** |  | **.171 [.035, .301], .014** |

**References**

Benjamini, Y., & Hochberg, Y. (1995). Controlling the False Discovery Rate: A Practical and Powerful Approach to Multiple Testing. *Journal of the Royal Statistical Society. Series B (Methodological)*, *57*, 289–300. http://doi.org/10.2307/2346101
