# Supplementary material for: Psychotic-Like Experiences at the Healthy End of the Psychosis Continuum
Source: Front Psychol. 2017 May 15;8:775. doi: 10.3389/fpsyg.2017.00775 (PMC5431212; doi:10.3389/fpsyg.2017.00775)
Supplement: Supplementary file 5 [file Table5.DOCX]

Supplementary Material

Psychotic-Like Experiences at the Healthy End of the Psychosis Continuum

Lui Unterrassner^1^*, Thomas Wyss^1^, Diana Wotruba^1^, Vladeta Ajdacic-Gross^2^, Helene Haker^1,3^, and Wulf Rössler^1,2,4^

*** Correspondence:** Corresponding Author: unterrassner@collegium.ethz.ch

**Supplementary Table 5**

**Correlation Matrix of Exceptional Experiences and PLE (Second Part).** Continued from Appendix 7. *r_s_* = Spearman’s rho; CI = confidence interval. The FDR corrected (Benjamini & Hochberg, 1995) alpha levels were .094 (.10, *trend*), .046 (.05, **significant**), and .008 (.01, **highly** **significant**).

|  |  |  |  | |  | |  |  |
| --- | --- | --- | --- | --- | --- | --- | --- | --- |
|  |  | *r_s_* [CI 95%], *p* | | | | | | |
|  |  |  |  |  | |  |  | |
| Item No. |  | SPQ  Unusual perceptual experiences |  | SPQ  Ideas of reference | |  | SPQ  Suspiciousness | |
| 01 |  | **.540 [.435, .630], .000** |  | **.309 [.180, .427], .000** | |  | **.287 [.157, .408], .000** | |
| 02 |  | **.309 [.180, .428], .000** |  | **.144 [.007, .275], .039** | |  | **.246 [.113, .370], .000** | |
| 03 |  | **.597 [.501, .678], .000** |  | **.433 [.315, .538], .000** | |  | **.274 [.142, .396], .000** | |
| 04 |  | **.404 [.283, .512], .000** |  | **.237 [.104, .362], .001** | |  | **.329 [.201, .445], .000** | |
| 05 |  | **.350 [.225, .465], .000** |  | **.354 [.229, .468], .000** | |  | **.316 [.187, .434], .000** | |
| 06 |  | **.381 [.257, .492], .000** |  | **.247 [.114, .371], .000** | |  | **.281 [.151, .403], .000** | |
| 07 |  | **.454 [.338, .556], .000** |  | **.382 [.259, .493], .000** | |  | **.214 [.080, .341], .002** | |
| 08 |  | **.310 [.181, .429], .000** |  | **.323 [.195, .440], .000** | |  | **.207 [.072, .334], .003** | |
| 09 |  | **.402 [.281, .511], .000** |  | **.277 [.145, .398], .000** | |  | **.213 [.079, .340], .002** | |
| 10 |  | **.466 [.352, .567], .000** |  | **.334 [.207, .450], .000** | |  | **.232 [.098, .357], .001** | |
| 11 |  | **.325 [.197, .442], .000** |  | **.230 [.096, .355], .001** | |  | **.178 [.042, .307], .011** | |
| 12 |  | **.469 [.355, .569], .000** |  | **.327 [.199, .444], .000** | |  | **.252 [.119, .375], .000** | |
| 13 |  | **.396 [.274, .505], .000** |  | **.318 [.189, .435], .000** | |  | **.169 [.033, .298], .015** | |
| 14 |  | **.452 [.337, .555], .000** |  | **.400 [.279, .509], .000** | |  | **.245 [.112, .370], .000** | |
| 15 |  | **.386 [.263, .496], .000** |  | **.242 [.109, .366], .000** | |  | **.190 [.054, .318], .006** | |
| 16 |  | **.369 [.245, .482], .000** |  | **.352 [.226, .466], .000** | |  | **.219 [.085, .345], .002** | |
| 17 |  | **.403 [.282, .511], .000** |  | **.274 [.143, .396], .000** | |  | **.142 [.006, .274], .041** | |
| 18 |  | **.426 [.307, .531], .000** |  | **.391 [.269, .501], .000** | |  | **.279 [.148, .400], .000** | |
| 19 |  | **.517 [.409, .610], .000** |  | **.454 [.339, .556], .000** | |  | **.287 [.157, .408], .000** | |
| 20 |  | **.413 [.293, .521], .000** |  | **.408 [.288, .516], .000** | |  | **.269 [.137, .391], .000** | |
| 21 |  | **.399 [.277, .508], .000** |  | **.199 [.064, .326], .004** | |  | **.201 [.066, .328], .004** | |
| 22 |  | **.429 [.311, .535], .000** |  | **.359 [.233, .472], .000** | |  | **.203 [.068, .330], .003** | |
| 23 |  | **.373 [.249, .485], .000** |  | **.338 [.211, .453], .000** | |  | **.267 [.135, .390], .000** | |
| 24 |  | **.284 [.153, .405], .000** |  | **.245 [.112, .369], .000** | |  | .059 [-.079, .194], .402 | |
| 25 |  | **.315 [.186, .433], .000** |  | **.25 [.119, .375], .000** | |  | **.177 [.041, .306], .011** | |
| 26 |  | **.416 [.296, .523], .000** |  | **.31 [.183, .430], .000** | |  | **.228 [.094, .353], .001** | |
| 27 |  | **.172 [.036, .301], .014** |  | **.17 [.041, .306], .011** | |  | .090 [-.048, .224], .200 | |
| 28 |  | **.366 [.242, .479], .000** |  | **.22 [.094, .354], .001** | |  | .063 [-.075, .198], .371 | |
| 29 |  | **.153 [.017, .284], .028** |  | **.17 [.037, .302], .013** | |  | **.175 [.040, .305], .012** | |
| 30 |  | **.245 [.112, .369], .000** |  | .09 [-.043, .228], .179 | |  | *.119 [-.018, .251], .089* | |
| 31 |  | *.119 [-.018, .252], .088* |  | .10 [-.031, .239], .129 | |  | -.029 [-.165, .108], .681 | |
| 32 |  | **.190 [.055, .319], .006** |  | **.17 [.040, .305], .011** | |  | .110 [-.027, .243], .114 | |

**References**

Benjamini, Y., & Hochberg, Y. (1995). Controlling the False Discovery Rate: A Practical and Powerful Approach to Multiple Testing. *Journal of the Royal Statistical Society. Series B (Methodological)*, *57*, 289–300. http://doi.org/10.2307/2346101
