# Supplementary material for: Psychotic-Like Experiences at the Healthy End of the Psychosis Continuum
Source: Front Psychol. 2017 May 15;8:775. doi: 10.3389/fpsyg.2017.00775 (PMC5431212; doi:10.3389/fpsyg.2017.00775)
Supplement: Supplementary file 6 [file Table6.docx]

Supplementary Material

Psychotic-Like Experiences at the Healthy End of the Psychosis Continuum

Lui Unterrassner^1^*, Thomas Wyss^1^, Diana Wotruba^1^, Vladeta Ajdacic-Gross^2^, Helene Haker^1,3^, and Wulf Rössler^1,2,4^

*** Correspondence:** Corresponding Author: unterrassner@collegium.ethz.ch

**Supplementary Table 6**

**Correlation Matrix of Psychotic-Like Experiences and Disorganized-, and Negative-Like Symptoms.** *r_s_* = Spearman’s rho; CI = confidence interval. The FDR corrected (Benjamini & Hochberg, 1995) alpha levels were .064 (.10, *trend*), .029 (.05, **significant**), and .005 (.01, **highly** **significant**).

|  |  |  |  | | | | |
| --- | --- | --- | --- | --- | --- | --- | --- |
|  |  |  | *r_s_* [CI 95%], *p* | | | | |
|  |  |  |  |  |  |  |  |
|  |  |  | Paranormal beliefs |  | Unusual perceptual experiences |  | Ideas of reference |
| Disorganized-like symptoms | |  |  |  |  |  |  |
|  | Odd speech |  | **.18 [.04, .31], .011** |  | **.34 [.21, .46], .000** |  | **.29 [.16, .41], .000** |
|  | Odd behaviour |  | **.20 [.07, .33], .004** |  | **.29 [.16 .41], .000** |  | **.38 [.26, .49], .000** |
|  |  |  |  |  |  |  |  |
| Negative-Like Symptoms | |  |  |  |  |  |  |
|  | Excessive social anxiety |  | .04 [-.10, .18], .547 |  | .05 [-.09, .19], .481 |  | **.18 [.04, .31], .012** |
|  | Constricted affect |  | -.06 [-.20, .08], .388 |  | .12 [-.02, .25], .090 |  | .07 [-.07, .20], .333 |
|  | No close friends |  | -.01 [-.15, .13], .860 |  | .08 [-.06, .21], .266 |  | .02 [-.12, .15], .816 |
|  | Physical Anhedonia Scale |  | **-.19 [-.32, -.05], .007** |  | -.06 [-.19, .08], .436 |  | -.07 [-.21, .06], .292 |
|  |  |  |  |  |  |  |  |
|  |  |  | Suspiciousness |  | Schizotypal signs |  | Schizophrenia nuclear symptoms |
| Disorganized-like symptoms | |  |  |  |  |  |  |
|  | Odd speech |  | **.35 [.23, .47], .000** |  | **.38 [.25, .49], .000** |  | *.13 [.00, .27], .055* |
|  | Odd behaviour |  | **.23 [.10, .36], .001** |  | **.36 [.24, .48], .000** |  | **.30 [.17, .42], .000** |
|  |  |  |  |  |  |  |  |
| Negative-Like Symptoms | |  |  |  |  |  |  |
|  | Excessive social anxiety |  | **.19 [.06, .32], .006** |  | **.30 [.17, .42], .000** |  | *.14 [.01, .28], .040* |
|  | Constricted affect |  | **.32 [.19, .44], .000** |  | **.35 [.23, .47], .000** |  | .04 [-.10, .17], .605 |
|  | No close friends |  | **.31 [.18, .43], .000** |  | **.28 [.15, .40], .000** |  | .11 [-.03, .25], .108 |
|  | Physical Anhedonia Scale |  | **.32 [.19, .44], .000** |  | **.22 [.09, .35], .001** |  | .02 [-.12, .16], .783 |

**References**

Benjamini, Y., & Hochberg, Y. (1995). Controlling the False Discovery Rate: A Practical and Powerful Approach to Multiple Testing. *Journal of the Royal Statistical Society. Series B (Methodological)*, *57*, 289–300. http://doi.org/10.2307/2346101
