# Supplementary material for: Psychotic-Like Experiences at the Healthy End of the Psychosis Continuum
Source: Front Psychol. 2017 May 15;8:775. doi: 10.3389/fpsyg.2017.00775 (PMC5431212; doi:10.3389/fpsyg.2017.00775)
Supplement: Supplementary file 7 [file DataSheet1.DOCX]

Supplementary Material

Psychotic-Like Experiences at the Healthy End of the Psychosis Continuum

Lui Unterrassner^1^*, Thomas Wyss^1^, Diana Wotruba^1^, Vladeta Ajdacic-Gross^2^, Helene Haker^1,3^, and Wulf Rössler^1,2,4^

*** Correspondence:** Corresponding Author: unterrassner@collegium.ethz.ch

**Supplementary Data 1**

1.1 **Factor analyses**

The majority (78%) of the PAGE-R items deviated from normal distributions (skewness and kurtosis exceeded absolute 1) and Mardia's test (Mardia, 1970) suggested excessive multivariate kurtosis in the data. Therefore, the use of a polychoric correlation matrix for the factor analysis was indicated (Muthén & Kaplan, 1985). The Kaiser–Meyer–Olkin value was 0.92, which exceeded the commonly recommended value of 0.6 and indicates suitability of the data for factor analysis. The Bartlett's test of sphericity reached statistical significance supporting the factorability of the correlation matrix (*p* = .000).

1.2 **Regression analyses**

Tests indicated that multicollinearity was not a concern (Tolerance > 0.1, VIF < 10) and the data met the assumptions of independent errors (Durbin-Watson value > 1 and < 3) and non-zero variances in all models. The scatterplot of standardized predicted values showed that the data met the assumptions of linearity and heteroscedasticity.

**References**

Mardia, K. V. (1970). Measures of multivariate skewness and kurtosis with applications. *Biometrika*, *57*(3), 519–530. http://doi.org/10.1093/biomet/57.3.519

Muthén, B., & Kaplan, D. (1985). A comparison of some methodologies for the factor analysis of non-normal Likert variables. *British Journal of Mathematical and Statistical Psychology*, *38*, 171–189. http://doi.org/10.1111/j.2044-8317.1992.tb00975.x
