# Supplementary material for: Psychotic-Like Experiences at the Healthy End of the Psychosis Continuum
Source: Front Psychol. 2017 May 15;8:775. doi: 10.3389/fpsyg.2017.00775 (PMC5431212; doi:10.3389/fpsyg.2017.00775)
Supplement: Supplementary file 8 [file DataSheet2.DOCX]

Supplementary Material

Psychotic-Like Experiences at the Healthy End of the Psychosis Continuum

Lui Unterrassner^1^*, Thomas Wyss^1^, Diana Wotruba^1^, Vladeta Ajdacic-Gross^2^, Helene Haker^1,3^, and Wulf Rössler^1,2,4^

*** Correspondence:** Corresponding Author: unterrassner@collegium.ethz.ch

**Supplementary Figure 1**

**Study Chart of the Exceptional Experiences Project.** Creative Experiences Questionnaire (Merckelbach, Horselenberg, & Muris, 2001), Childhood Trauma Questionnaire (Bernstein & Fink, 1998), Competence and Locus of Control (Krampen, 1991), Handedness (J. P. Chapman & Chapman, 1987), Morningness-Eveningness Questionnaire (Horne & Ostberg, 1976), Magical Ideation Scale (Eckblad & Chapman, 1983), Optimistic Bias Scale (Tamagni et al., 2010), Revised Exceptional Experiences Questionnaire (Fach, Atmanspacher, Landolt, Wyss, & Rössler, 2013), Physical Anhedonia Scale (L. J. Chapman, Chapman, & Raulin, 1976), Religion - Belief – Spirituality (Huber, 2008), Revised Symptom Checklist 90 (Derogatis, 1977), Schizotypal Personality Questionnaire (Raine, 1991), Self Report on Health Status (Campbell, 2000), Sense of Coherence Scale (Antonovsky, 1993).

**References**

Antonovsky, A. (1993). the Structure and Properties of the Coherence Sense. *Sot. Sri. Med.*, *36*(6), 125–733. http://doi.org/10.1016/0277-9536(93)90033-z

Bernstein, D., & Fink, L. (1998). Manual for the childhood trauma questionnaire. *The Psychological Corporation*.

Campbell, J. J. (2000). Neuropsychiatric assessment. In C. Coffey & E. Cumming (Eds.), *Textbook of Geriatric Neuropsychiatry* (2nd ed., pp. 109–124). Washington DC: American Psychiatric Press.

Chapman, J. P., & Chapman, L. J. (1987). Handedness of hypothetically psychosis-prone subjects. *Journal of Abnormal Psychology*, *96*(2), 89–93. http://doi.org/10.1037/0021-843X.96.2.89

Chapman, L. J., Chapman, J. P., & Raulin, M. L. (1976). Scales for physical and social anhedonia. *Journal of Abnormal Psychology*, *85*(4), 374–382. http://doi.org/10.1037/0021-843X.85.4.374

Derogatis, L. R. (1977). *SCL-90. Administration, Scoring and Procedures Manual-1 for the R (Revised) Version and Other Instruments of the Psychopathology Rating Scale Series*. Baltimore MD: Johns Hopkins University Press.

Eckblad, M., & Chapman, L. J. (1983). Magical ideation as an indicator of schizotypy. *Journal of Consulting and Clinical Psychology*, *51*(2), 215–225. http://doi.org/10.1037/0022-006X.51.2.215

Fach, W., Atmanspacher, H., Landolt, K., Wyss, T., & Rössler, W. (2013). A comparative study of exceptional experiences of clients seeking advice and of subjects in an ordinary population. *Frontiers in Psychology*, *4*(February), 65. http://doi.org/10.3389/fpsyg.2013.00065

Horne, J. a., & Ostberg, O. (1976). A self-assessment questionnaire to determine morningness-eveningness in human circadian rhythms. *Int J Chronobiol*. http://doi.org/10.1177/0748730405285278

Huber, S. (2008). Kerndimensionen, Zentralität und Inhalt. Ein interdisziplinäres Modell der Religiosität. *Journal Für Psychologie*, *16*(3), 1–17. Retrieved from http://www.journal-fuer-psychologie.de/index.php/jfp/article/view/202

Krampen, G. (1991). *Fragebogen zu Kompetenz- und Kontrollüberzeugungen*. *(FKK)*.

Merckelbach, H., Horselenberg, R., & Muris, P. (2001). The Creative Experiences Questionnaire (CEQ): A brief self-report measure of fantasy proneness. *Personality and Individual Differences*, *31*(6), 987–995. http://doi.org/10.1016/S0191-8869(00)00201-4

Raine, A. (1991). The SPQ: a scale for the assessment of schizotypal personality based on DSM-III-R criteria. *Schizophrenia Bulletin*, *17*, 555–564. http://doi.org/10.1093/schbul/17.4.555

Tamagni, C., Palla, A., Krummenacher, P., Vitacco, D., Huberle, E., Straumann, D., … Brugger, P. (2010). *Vestibular stimulation reduces unrealistic optimism*. *Psychiatry: Interpersonal and Biological Processes*. Retrieved from http://precedings.nature.com/documents/4519/version/1/files/npre20104519-1.pdf
