# Supplementary figures and images for: Psychotic-Like Experiences at the Healthy End of the Psychosis Continuum
Source: Front Psychol. 2017 May 15;8:775. doi: 10.3389/fpsyg.2017.00775 (PMC5431212; doi:10.3389/fpsyg.2017.00775)

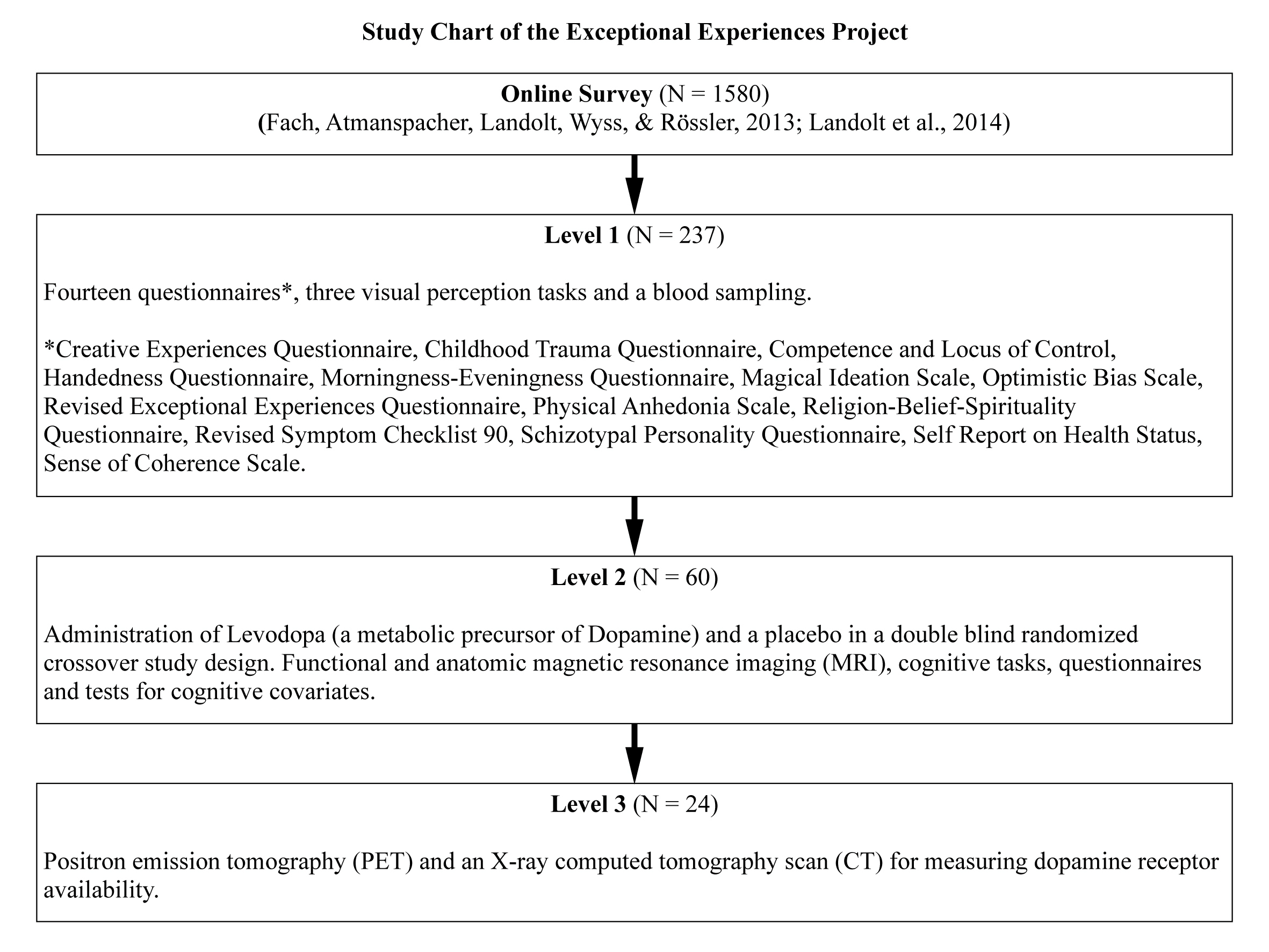

Supplement: Supplementary file 10 [file Image1.JPEG]

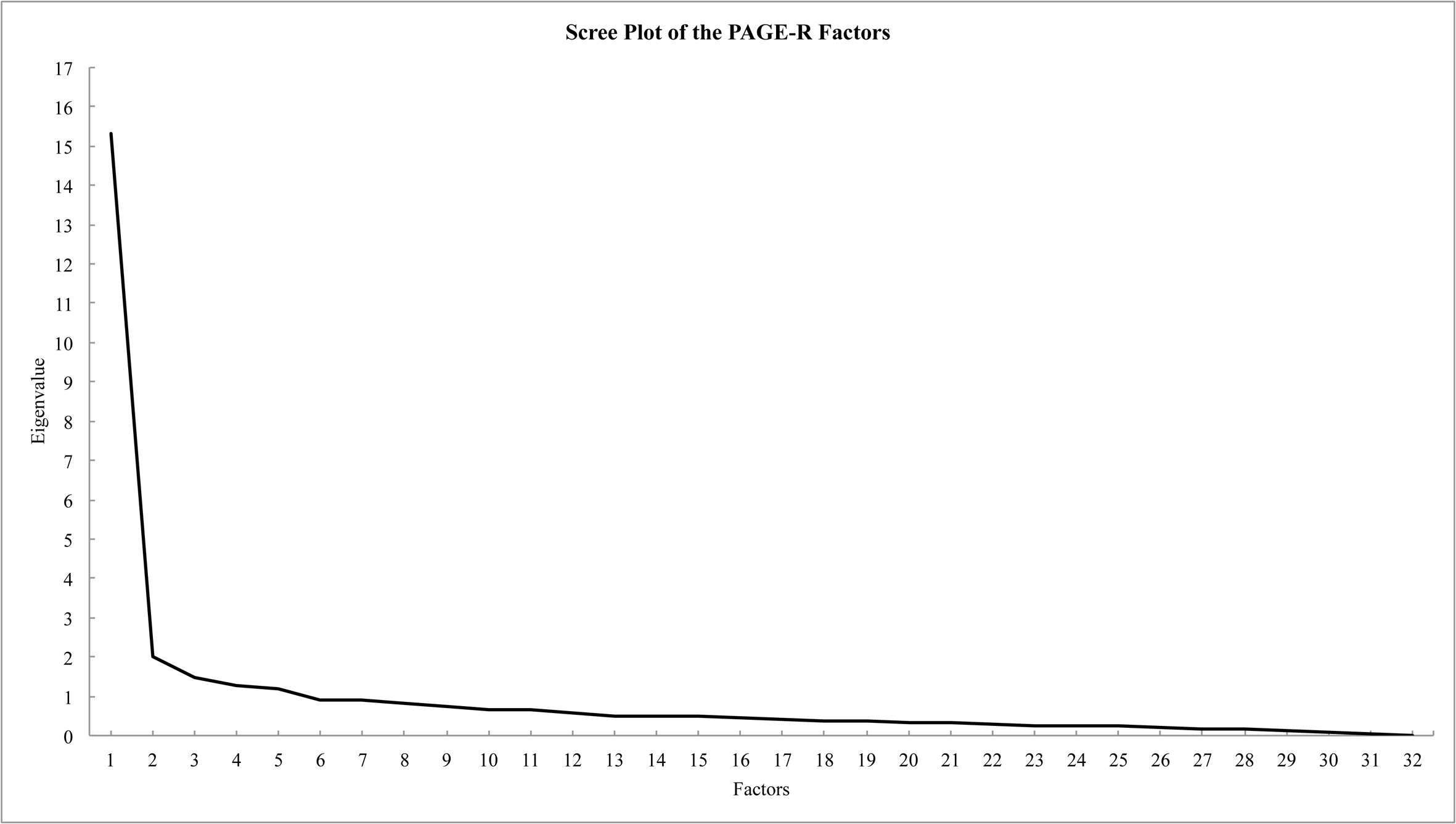

Supplement: Supplementary file 11 [file Image2.JPEG]
